# Gene therapy for infantile malignant osteopetrosis: review of pre-clinical research and proof-of-concept for phenotypic reversal

**DOI:** 10.1016/j.omtm.2020.12.009

**Published:** 2020-12-25

**Authors:** Ilana Moscatelli, Elena Almarza, Axel Schambach, David Ricks, Ansgar Schulz, Christopher D. Herzog, Kim Henriksen, Maria Askmyr, Jonathan D. Schwartz, Johan Richter

**Affiliations:** 1 Department of Molecular Medicine and Gene Therapy, Lund Strategic Center for Stem Cell Biology, Lund University, Lund, Sweden; 2 Rocket Pharmaceuticals, Inc., New York, NY, USA; 3 Institute of Experimental Hematology, Hannover Medical School, Hannover, Germany; 4 Division of Hematology/Oncology, Boston Children’s Hospital, Harvard Medical School, Boston, MA, USA; 5 Department of Pediatrics and Adolescent Medicine, University Medical Center Ulm, Germany; 6 Nordic Bioscience A/S, Herlev, Denmark

**Keywords:** infantile malignant osteopetrosis, lentivirus, gene therapy, autosomal recessive osteopetrosis, hematopoietic stem and progenitor cells, osteoclast disorders

## Abstract

Infantile malignant osteopetrosis is a devastating disorder of early childhood that is frequently fatal and for which there are only limited therapeutic options. Gene therapy utilizing autologous hematopoietic stem and progenitor cells represents a potentially advantageous therapeutic alternative for this multisystemic disease. Gene therapy can be performed relatively rapidly following diagnosis, will not result in graft versus host disease, and may also have potential for reduced incidences of other transplant-related complications. In this review, we have summarized the past sixteen years of research aimed at developing a gene therapy for infantile malignant osteopetrosis; these efforts have culminated in the first clinical trial employing lentiviral-mediated delivery of *TCIRG1* in autologous hematopoietic stem and progenitor cells.

## Graphical Abstract

**Figure:**
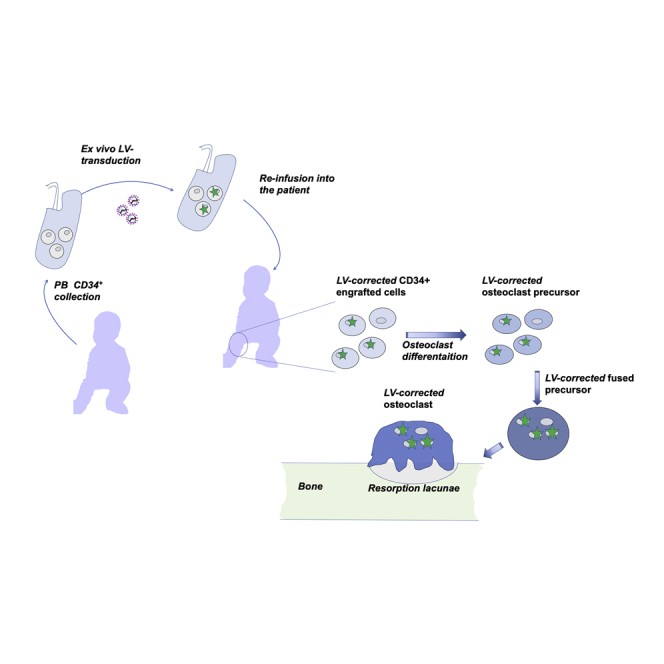

## Teaser

Infantile malignant osteopetrosis (IMO) presents a highly unmet medical need with limited therapeutic options. Gene therapy utilizing autologous hematopoietic stem and progenitor cells represents a potentially advantageous therapeutic alternative for this disease. Here, we have summarized all nonclinical studies supporting the initiation of a clinical gene therapy trial for IMO.

## Main text

### Infantile malignant osteopetrosis (IMO)

**Figure 1:**
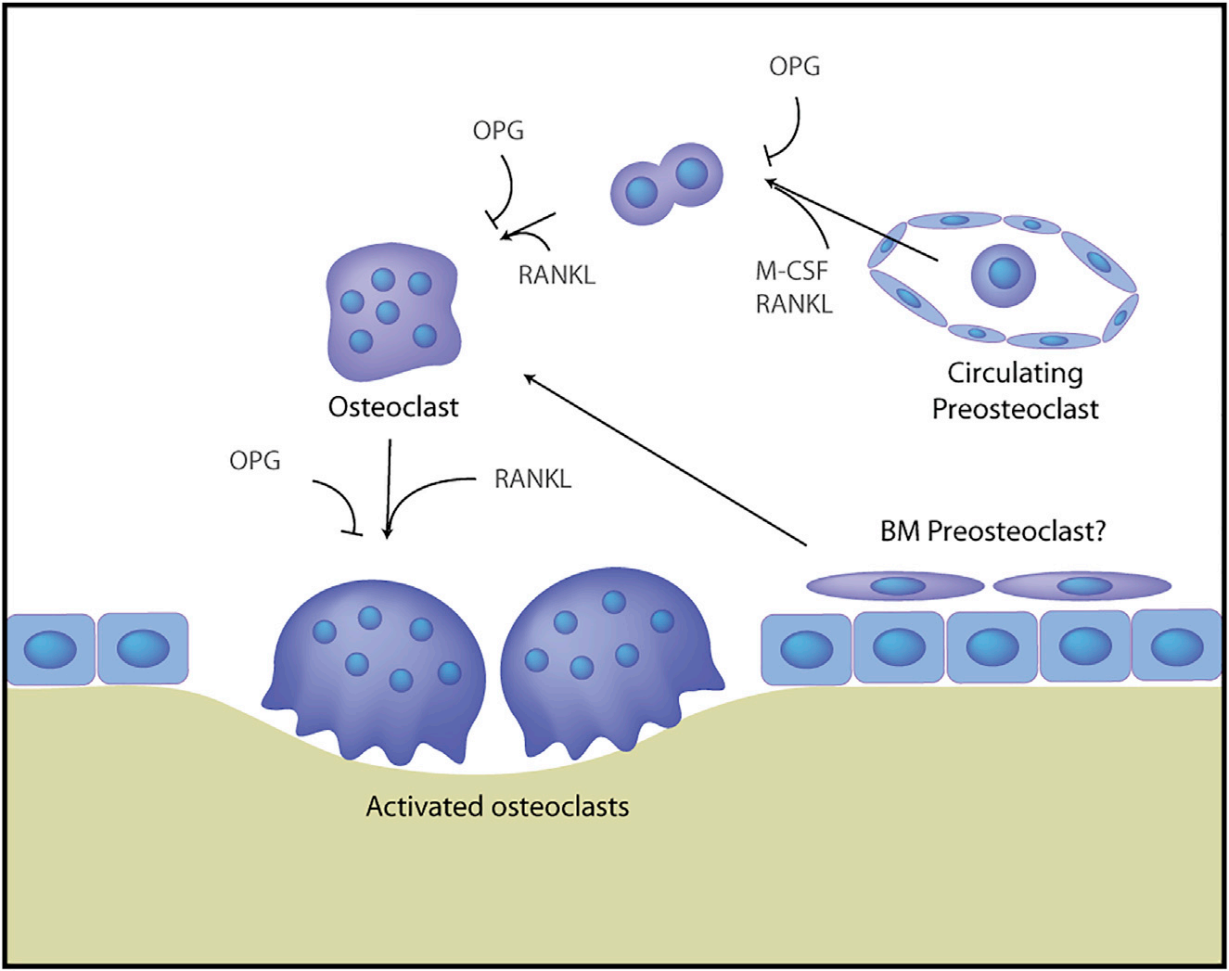
Osteoclast differentiation pathways Osteoclasts are differentiated from peripheral blood monocytic cells and possibly also from quiescent precursors in the bone^2^. M-CSF, macrophage colony-stimulating factor; RANKL, receptor activator of nuclear factor kB ligand; OPG, osteoprotegrin; BM, bone marrow.

Osteopetrosis is a group of skeletal genetic disorders characterized by altered bone mass due to defects in osteoclast development and/or function. Bone growth and homeostasis are dependent upon a continuous equilibrium between bone formation and resorption. This equilibrium predominantly relies on two cell types: osteoblasts, derived from mesenchymal progenitors and responsible for bone formation; and osteoclasts, large and multinucleated cells derived from the myeloid lineage and responsible for bone resorption. Bone resorption is essential for the maintenance of calcium levels in the blood, fracture healing, tooth eruption, and skeletal modeling during growth or repair of bone micro-damage from routine stress.^1^ Osteoclast differentiation occurs when progenitors of the monocytic lineage exit the bloodstream, aggregate at sites of bone resorption, and fuse to form these large and multinucleated cells (Figure 1).

*TCIRG1*-mediated IMO, also known as autosomal recessive osteopetrosis (ARO), is a very rare pediatric disorder that confers significant morbidity and mortality during the first decade of life. IMO is estimated to affect approximately 1/200,000 live births, although a higher incidence has been observed in selected geographic regions, most notably Costa Rica.^3^ As described above, the underlying defect is impaired bone resorption secondary to deficient osteoclast function. IMO patients present with bone abnormalities, including dense and brittle bones, and neurologic abnormalities including early and rapidly progressive vision loss due to impingement of cranial nerve foramina. Hypocalcemia and secondary hypoparathyroidism are observed frequently. Bone marrow failure (BMF) is progressive and a frequent cause of mortality; hepatosplenomegaly occurs frequently as a consequence of extramedullary hematopoiesis.^4^ In the absence of definitive therapy, IMO is frequently fatal within the initial two years of life and affected children rarely survive more than 10 years; those that do frequently suffer from extensive morbidity.^5^^,^^6^

**Figure 2:**
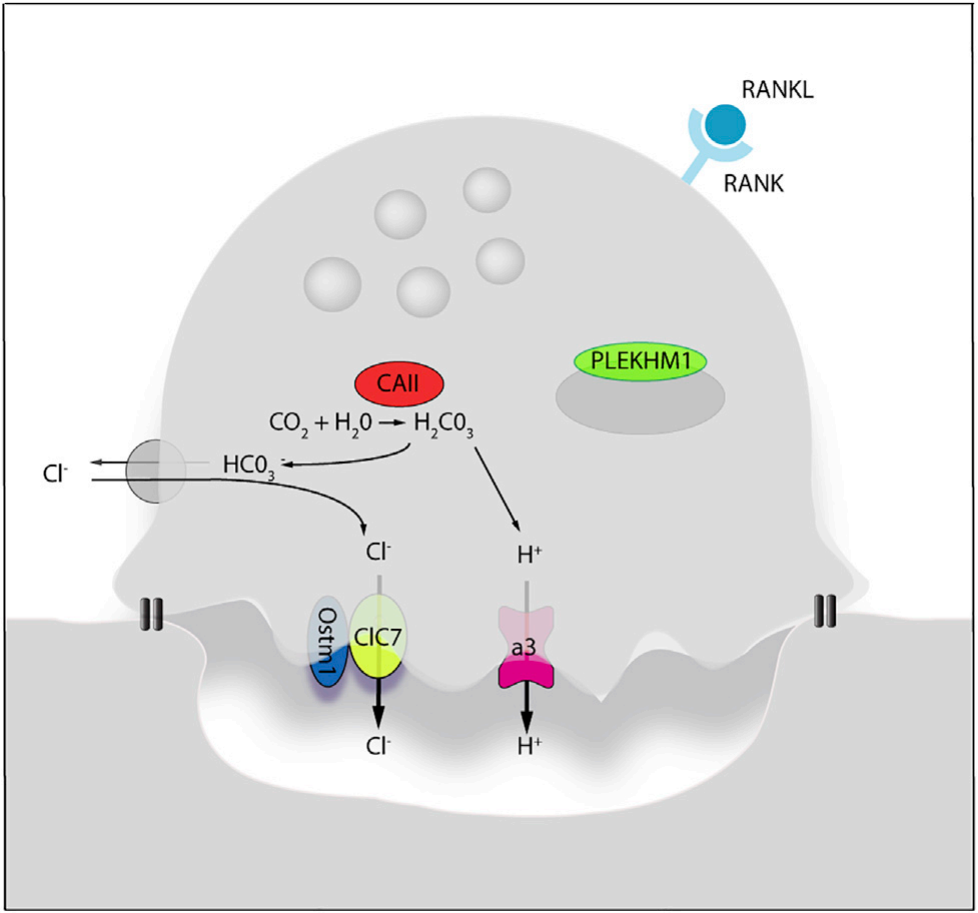
Activated osteoclast function Human osteopetrosis is mainly caused by mutations in genes that code for highlighted proteins in the figure, that are involved in acidification *lacuna.*^2^ More than 50% of cases of recessive infantile malignant osteopetrosis (IMO) are due to mutations in *TCIRG1* (that codes for a3 proton pump in the figure). Other genes involved in IMO are *CLCN7* (affecting chloride channel 7) and *OSTM1*, which colocalizes with CLCN7 and is involved in regulation of its activity. CAII, carbonic anhydrase II; PLEKHM1, pleckstrin homology domain-containing family M (with RUN domain) member I; OSTM1; osteopetrosis associated transmembrane protein 1.

Mutations in at least 10 known genes have been associated with an osteopetrotic phenotype^7^ and the most frequent are those in the T cell immune regulator 1 (*TCIRG1*) gene, accounting for approximately 50% of autosomal recessive IMO cases.^8^, ^9^, ^10^ The *TCIRG1* gene is located on chromosome 11q13.4-q13.5^11^ and gives rise to two alternatively spliced products, TIRC7 and OC116. TIRC7 is a T cell membrane protein essential for T cell activation. OC116 is the 116 kDa a3 subunit of the vacuolar V-ATPase proton pump that mediates H^+^ transport into the resorption area beneath the osteoclast and is responsible for acidification of endosomes and lysosomes (Figure 2).^12^, ^13^, ^14^ Acidified endosomes and lysosomes enable formation of a functional osteoclast ruffled border essential for bone resorption, an integral component of normal bone and marrow growth and development. Mutations in other genes have also been associated with clinical IMO, and a correlation between affected gene and phenotype has been established.^15^ Mutations on the *CLCN7* gene account for the second-most-frequent form (17% of IMO cases) and are responsible for a wide spectrum of clinical manifestations, including primary neurodegeneration and hematologic failure. Mutations in *OSTM1* account for approximately 5% of IMO cases and are associated with severe primary neurodegeneration and a reduced life expectancy (2 years).^6^^,^^15^

### Treatments for IMO

Treatment for IMO is primarily palliative and directed at symptomatic management of complications. The disease often progresses rapidly during the initial years of life, and rapid initiation of definitive treatment after diagnosis is essential to minimize morbidity and mortality. Despite advances in supportive care, BMF is a particularly devastating and fatal consequence of the disease in the absence of successful allogeneic hematopoietic stem cell transplantation (HSCT). Because osteoclasts derive from the myeloid lineage, HSCT represents a potentially definitive treatment.^16^, ^17^, ^18^ Outcomes of HSCT in IMO patients have shown an estimated 88% survival for recipients of HLA-identical HSCT, 80% survival for recipients of HLA-matched HSCT, and 66% for patients receiving HLA-haplotype-mismatched HSCT.^15^ Although HSCT may be curative, overall survival following transplant has historically been lower than in some other non-malignant hematologic disorders, particularly if no HLA-identical donor is available, as a result of the frequent short- and long-term transplant complications including primary and secondary graft failure, pneumonitis, veno-occlusive disease (VOD), and graft-versus-host disease (GvHD).^19^^,^^20^ Due to a very high risk of progressive blindness, neurodegeneration, and BMF in the absence of definitive therapy, IMO represents a high unmet medical need for which there are limited therapeutic options.

### Gene therapy for non-malignant hematopoietic disorders

Over the last 20 years, hematopoietic cell-based gene therapy (GT) has advanced both in terms of safety and efficacy. The initial GT clinical trials for hematopoietic disorders were conducted in the 1990s and utilized long terminal repeat (LTR)-driven γ-retroviral vectors (γRVs) aimed at gene-based correction of functional defects underlying several primary immunodeficiencies, including X-linked severe combined immunodeficiency (SCID-X1),^21^, ^22^, ^23^, ^24^ adenosine deaminase (ADA)-SCID,^25^, ^26^, ^27^ chronic granulomatous disease (CGD),^28^^,^^29^ and Wiskott-Aldrich syndrome (WAS).^30^^,^^31^ Unfortunately, insertional mutagenic events were detected in several of these trials,^21^^,^^31^, ^32^, ^33^, ^34^, ^35^, ^36^ with the exception of ADA-SCID.^37^ These LTR-driven γRVs were shown to have a propensity for integration in proximity to proto-oncogenes (especially *LMO2* and *EVI1*) and promoted their transactivation through the proviral LTR enhancer. Both the γRV integration profile (with preferential integration close to the transcription start sites of actively transcribed genes^38^) and the configuration of these vectors (in which potent U3 enhancer sequences within the LTRs were present in the integrated provirus) are believed to have contributed to the frequency of insertional mutagenesis.

Subsequent to these initial γRV-based studies, there has been a focus on the development of potentially safer self-inactivating (SIN) vectors, in which the enhancer regions that caused transactivation in previous trials were eliminated from the integrated provirus. A SIN γRV was developed for the treatment of X1-SCID that showed efficacy and improved safety.^39^ Additional efforts were conducted to develop SIN lentiviral vectors (LVs), with safer, less *cis*-acting vector architecture and a potentially safer integrome.^40^ As of August 2020, evidence of clinical efficacy has been demonstrated in more than 200 patients collectively in Europe and the US who have received investigational therapy with autologous hematopoietic stem cells transduced with LV (FDA Workshop 2020: Facilitating End-to-End Development of Individualized Therapeutics), including patients with Fanconi anemia (FA), SCID, beta thalassemia, sickle cell anemia, WAS, CGD, childhood cerebral adrenoleukodystrophy, metachromatic leukodystrophy, and leukocyte adhesion deficiency type I (LAD-I). To date, with a follow-up through 10 years for the earliest treated patients, no instances of insertional mutagenesis, vector-related myelodysplasia/acute leukemia, or replication-competent lentivirus (RCL) transmission have been reported for patients in these clinical studies.^41^, ^42^, ^43^, ^44^, ^45^, ^46^, ^47^

### GT for IMO

Based on this growing body of safety data, GT utilizing autologous hematopoietic stem and progenitor cells (HSPCs) therefore represents a potentially safe and promising therapeutic alternative for IMO. GT can be implemented relatively rapidly following diagnosis and does not require identification of a suitable allogeneic donor. In IMO patients, autologous GT will not lead to GvHD and may have potential for reduced incidences of other HSCT-related pulmonary and hepatic complications. GT entails collection of HSPCs from peripheral blood (PB; after mobilization from the BM or other hematopoietic organs), *ex vivo* HSPC transduction and re-infusion of corrected gene-expressing cells into patients. Engrafted HSPCs are expected to generate multiple types of mature blood cells, including osteoclasts developing from the monocytic lineage.^48^

The availability of HSPCs in IMO-affected children is an important consideration for the feasibility of IMO GT. Extraction of sufficient HSPC quantities from PB following mobilization is a vital component to enable this GT approach. Although IMO-afflicted children have reduced BM cavities limiting feasibility of BM HSPC extraction, levels of circulating PB HSPCs are markedly increased in IMO patients^49^^,^^50^ and adequate collection of HSPCs is therefore expected. Nevertheless, due to rapid disease progression, expedient initiation of HSPC collection and subsequent treatment are essential.

### Extent of hematopoietic correction required for phenotypic correction

**Figure 3:**
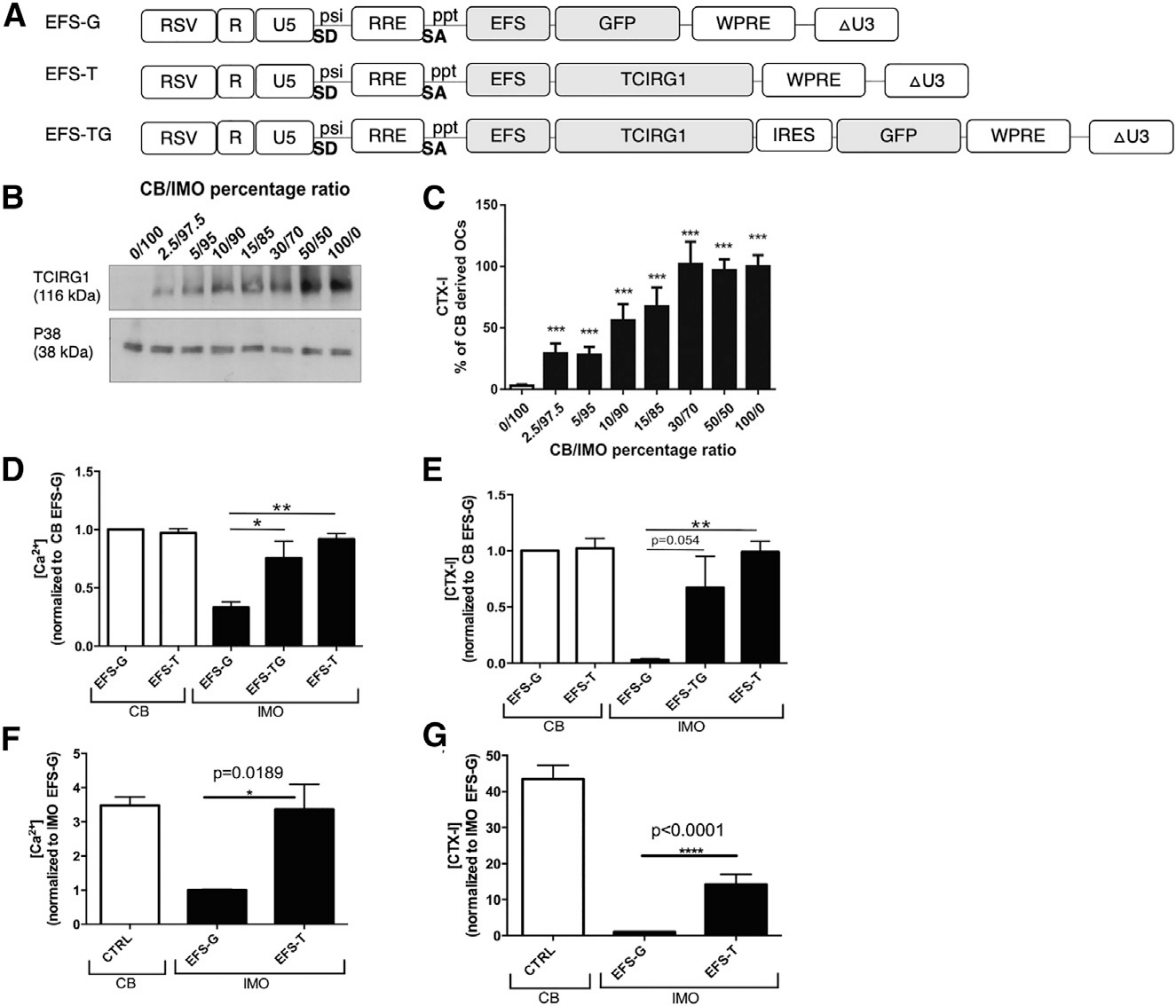
Correction of IMO phenotype in human cells (A) Vector constructs used in experiments. (B and C) Corrective effect of mixing normal cord blood (CB) and IMO cells.^52^ In (B), protein expression of TCIRG1 was analyzed in lysed cells by western blot and p38 was used as loading control. Data in (C) are expressed as values compared to those obtained from osteoclasts generated from 100% CB CD34^+^ cells. Statistical analysis was done using one-way ANOVA with a Dunnett post-test comparing to 100% IMO CD34^+^ cells. (D and E) Clinically relevant EFS.hTCIRG1-LV vector (corresponds with EFS-T in the figure) can restore resorption in osteoclasts differentiated from IMO CD34^+^ cells *in vitro*. Transduced cells were expanded for 2 weeks, seeded on bone slices, and differentiated into osteoclasts for 13 days in the presence of M-CSF and RANKL. The data are shown as means ± SEM.^53^ (F and G) Vector-corrected IMO osteoclasts generated from NSG-engrafting hematopoietic cells show rescued resorption capacity. Bone-marrow cells were harvested from NSG mice 9–19 weeks after transplantation. Human CD34^+^ cells were expanded for 2 weeks, seeded on bone slices, and differentiated into osteoclasts for 13 days in the presence of M-CSF and RANKL. The data are shown as means ± SEM.^53^ EFS, elongation factor 1α short; ppt, central polypurine tract; RSV, Rous sarcoma virus promoter; SA, splice acceptor site; SD, splice donor site; psi, packaging signal; CTX-I, C-terminal type I collagen fragment. Statistical significance is indicated with *p < 0.05, **p < 0.01, ***p<0.001 and ****p<0.0001.

An additional important consideration is the level of corrected osteoclasts likely required to confer clinical benefit. The relationship between the degree of HSPC correction and functional benefit has been gleaned from nonclinical studies. Initial *in vivo* observations in neonatal *oc/oc* mice receiving transplantation of wild-type cells without prior conditioning resulted in more than 85% survival of transplanted mice and correction of bone phenotype despite the presence of only 3%–5% engrafted cells.^51^ *In vitro* mixing studies using osteoclasts derived from IMO patients and healthy donor cord blood (CB) CD34^+^ cells also revealed that even low levels (2.5%) of non-IMO CD34^+^ cells, when mixed with IMO cells and differentiated to osteoclasts *ex vivo*, resulted in detectable TCIRG1 protein expression (Figure 3B) and significantly increased *ex vivo* bone resorption as assayed by C-terminal type I collagen fragment (CTX-I) release *in vitro*. The presence of 10% CB-derived osteoclasts resulted in more than 50% of normal resorption, and 30% CB-derived osteoclasts resulted in normal resorption levels^52^ (Figure 3C). Marked increases in *ex vivo* resorption were also observed when IMO-derived CD34^+^ cells were mixed with LV-corrected counterparts and similarly differentiated. Multiple studies have indicated that CTX-I levels are a highly relevant measurement of bone resorption *in vitro* as they correlate well to the area and volume of resorbed bone on cortical bovine bone slices in studies involving osteoclasts generated from osteopetrosis patients;^54^, ^55^, ^56^ these include studies involving inhibitors of osteoclast acid secretion^57^, ^58^, ^59^, ^60^ and studies of glucocorticoid-induced bone resorption.^61^ In aggregate, these studies indicate that even modest levels of HSPC correction may confer phenotypic reversal and therapeutic benefit in IMO patients.

### Induced pluripotent stem cells as a potential mechanism for GT

Additional efforts have been made to develop a source of pre-osteoclasts as a potential supportive cellular therapy. Human iPSCs (induced pluripotent stem cells) from healthy fibroblasts can be differentiated into monocytes that will express pre-osteoclast markers utilizing a differentiation protocol in a pre-defined media and in the presence of specific cytokines.^62^ Cells with a multinucleated phenotype can be further generated in the presence of macrophage colony-stimulating factor (M-CSF) and receptor activator of nuclear factor kB ligand (RANKL) factors. These cells exhibiting an osteoclast phenotype were shown to be capable of effective *in vitro* bone resorption.^63^ iPSCs can also be generated from IMO patient-derived cells and, following the same protocol, it is possible to obtain IMO-derived differentiated osteoclasts with strongly impaired resorption. Gene correction of these iPSCs with gamma-RV^64^ or LV^63^ containing non-mutated *TCIRG1* resulted in differentiated osteoclasts with restored ability to acidify resorption lacunae and resorb bone *in vitro*. Additional studies of gene correction in IMO-derived iPSCs were carried out by homologous recombination strategies. iPSCs generated from *oc/oc* mice were corrected with a BAC carrying the entire *Tcirg1* gene locus and differentiated into osteoclasts with rescued bone resorption capacity.^65^ In humans, these autologous iPSCs could potentially provide an unlimited source of pre-osteoclasts to support the early phase of recovery after transplantation and it has been proposed that IMO patients could also benefit from early transplantation of myeloid progenitors differentiated toward the osteoclast lineage.^66^^,^^67^

### Mouse models of osteopetrosis

Several spontaneous and genetically modified osteopetrotic mutant mouse models have been developed during recent decades in which osteoclasts are either absent or nonfunctional. These models have contributed greatly to knowledge regarding osteoclast differentiation, activity, and function. Of these models, the *oc/oc*, *Clcn7*^*−/−*^, and *gl/gl* mice carry mutations in the genes with the highest incidence in human IMO (*Tcirg1*, *Clcn7*, and *Ostm1*, respectively) and thus most substantially facilitated study of the pathophysiology of the most severe forms of human osteopetrosis.^15^

The *oc/oc* mouse model harbors a deletion mutation in the *Tcirg1* gene and displays characteristics of the human disease, supporting its utility as an animal model. This mouse model contains a *Tcirg1* mutation resulting in absent expression of the a3 subunit of the vacuolar H(+)-ATPase. As in human IMO, this protein isoform is involved in pH regulation of intracellular compartments and organelles including osteoclast resorption *lacunae*, and its absence leads to the formation of *oc/oc* osteoclasts incapable of bone resorption. The *oc/oc* mice develop an osteopetrotic pathophysiology and phenotype that replicates several of the most salient aspects of the human disorder: growth retardation, skeletal abnormalities, absence of teeth, blindness, encroachment of marrow cavities leading to BMF, and severely reduced lifespan.^68^^,^^69^

Additional efforts have been conducted during recent years by several groups to generate a mouse model that both displays an osteopetrotic phenotype and may host engraftment of transplanted human HSPCs. *Oc/oc* mice are immunocompetent and will reject transplanted human HSPCs without additional manipulation. Additionally, human HSPCs will not differentiate into monocytes, macrophages, or osteoclasts in a mouse model unless human M-CSF is present (i.e., murine M-CSF does not stimulate the human receptor). Montano and collaborators at Lund University (C. Montano, I. Moscatelli, C. Flores, C. Thudium, A. Schulz, A. Schambach, K. Henriksen, J. Richter, 2013, ESGCT conference) focused their efforts during 2011–2015 to develop an NSG/*oc/oc* mouse strain crossing *oc/+* mice (C57BL/6J × C3HheB/Fej) with the NSG mouse population (NOD-SCID interleukin-2rγ^null^ [IL-2rγ^null^]). However, the NSG*/oc/oc* mice to a large extent died in utero or within the initial days of life, preventing any opportunities for additional manipulation or evaluation. Similar efforts during 2018–2019 yielded equally limited results (Transgenic Core Facility, University of Copenhagen). A very recent publication detailed the successful generation of an NSG/*oc/oc* mouse model that involved backcrossing the *oc/+* mouse (B6C3Fe a/a Tcirg1 oc/J-Ly5.2) on the C57BL/6J background followed by cross-breeding with the NSG mouse (NOD-SCID IL-2rγ^null^) to enable an F1 generation capable of breeding NSG/*oc/oc* offspring.^70^ In addition to the above mentioned phenotypic characteristics of the oc/oc mouse, splenomegaly was observed in this novel model. NSG *oc/oc* mice showed human chimerism when transplanted with human CD34^+^ cells although the bone of transplanted NSG/*oc/oc* mice remained osteopetrotic despite administration of human M-CSF. Future xenotransplant experiments utilizing this mouse model could be of potential value if the appropriate conditions are identified, including the use of human cytokines or further crossing with another genetic background, to allow human osteoclast differentiation.

### Proof-of-concept for GT in IMO with γRV

Transplantation experiments performed in the *oc/oc* mouse model demonstrated that the osteopetrotic phenotype of these mice could be reversed by neonatal and in utero HSCT using wild-type cells.^71^^,^^72^ The first demonstrated proof-of-concept for the GT of IMO utilized γRV and the *oc/oc* mouse model. HSPCs from *oc/oc* mice were transduced with a *Tcirg1-*γRV (a gamma retroviral backbone in which *Tcirg1* transgene expression was driven by a spleen focus-forming virus [SFFV] LTR) and transplanted into irradiated neonatal *oc/oc* mice. Approximately 50% of treated mice achieved long-term survival,^73^ with an engraftment level of approximately 20% of corrected cells. BM cells were harvested from treated mice and differentiated *in vitro*, with resultant osteoclasts arising in both normal quantity and morphology. Bone resorbing capacity of these cells was approximately 10% compared to osteoclasts generated from wild-type BM cells; however, there was an almost complete normalization of the skeletal phenotype in mice surviving long-term. These results indicate that a healthy balance between production and destruction of bone may be achieved with only a modest proportion of osteoclasts with resorptive capacity. These results provide additional data—in the context of a strong viral promoter—indicating that even modest engraftment levels of gene-corrected cells may result in transgene expression levels sufficient to correct the most serious osteopetrosis-related manifestations.

### Development of clinically applicable LV for the treatment of IMO

As described previously, subsequent to the initial GT clinical trials performed with γRV, the GT field has incorporated the use of SIN-LV with a safer integration profile. Following the initial experience with γRVs, the first proof-of-concept experiments with LV were performed with the same strong SFFV promoter as above (as an internal promoter) to drive *hTCIRG1* expression along with the marker gene green fluorescent protein (GFP) co-expressed via an internal ribosomal entry site (IRES; SFFV.hTCIRG1-GFP-LV). Transduction of IMO CD34^+^ cells with SFFV.hTCIRG1-GFP-LV resulted in complete restoration of derived osteoclast resorptive function, as measured by CTX-I release *in vitro.*^56^ Similarly to results observed with CB mixing experiments, the presence of approximately 30% osteoclasts derived from LV-transduced IMO cells was sufficient to fully restore bone resorption capacity *in vitro*. However, because the SFFV promoter is not clinically applicable due to risk of insertional mutagenesis,^74^ two subsequent vectors with more physiologically relevant mammalian promoters were generated and tested. These newly evaluated promoters were the elongation factor 1α short (EFS) promoter, which drives ubiquitous transgene expression,^75^^,^^76^ and the chimeric myeloid (ChimP) promoter, which confers preferential myeloid transgene expression.^77^ *In vitro* studies using osteoclasts derived from LV-transduced IMO cells showed that the EFS promoter was the most efficient in terms of both protein expression and bone resorption parameters. A final clinical candidate LV (EFS.hTCIRG1-LV) was generated by removing the IRES- and GFP-encoding sequences from the vector mentioned above. *In vitro* studies demonstrated that this LV construct (Figure 3A) almost completely restored resorptive function (>90%) of osteoclasts derived from CD34^+^ cells from patients with IMO, as shown by release of Ca^2+^ and CTX-I, when compared to osteoclasts derived from healthy CB CD34^+^ cells (Figures 3D and 3E).^53^

After these initial *in vitro* results with the EFS.hTCIRG1-LV, efforts were subsequently directed toward the demonstration of phenotypic correction in IMO human cells using a xenograft transplantation model (immunodeficient NSG mice). LV transduced CD34^+^ cells from the PB of IMO patients showed similar engraftment levels in NSG mice as healthy donor CB-derived CD34^+^ cells without any indication of hematopoietic lineage perturbation. Analyses of recipients’ BM revealed human engraftment of approximately 35% with no differences observed in lineage distribution of BM cells harvested from mice transplanted with IMO CD34^+^ cells transduced with EFS.hTCIRG1-LV, as compared to EFS.GFP-LV, indicating that EFS.hTCIRG1-LV does not alter the differentiation potential of transduced IMO CD34^+^ cells in this model. Because human osteoclasts do not respond to murine Csf1, they do not develop in murine models. Human CD34^+^ cells were therefore harvested from the BM of transplanted animals and subsequently differentiated into osteoclasts on bovine bone slices *ex vivo.* Evaluation of bone resorption showed 33% of normal activity (based on CTX-I release, Figure 3G) and potentially complete restoration (based on calcium release, Figure 3F).^53^

**Figure 4:**
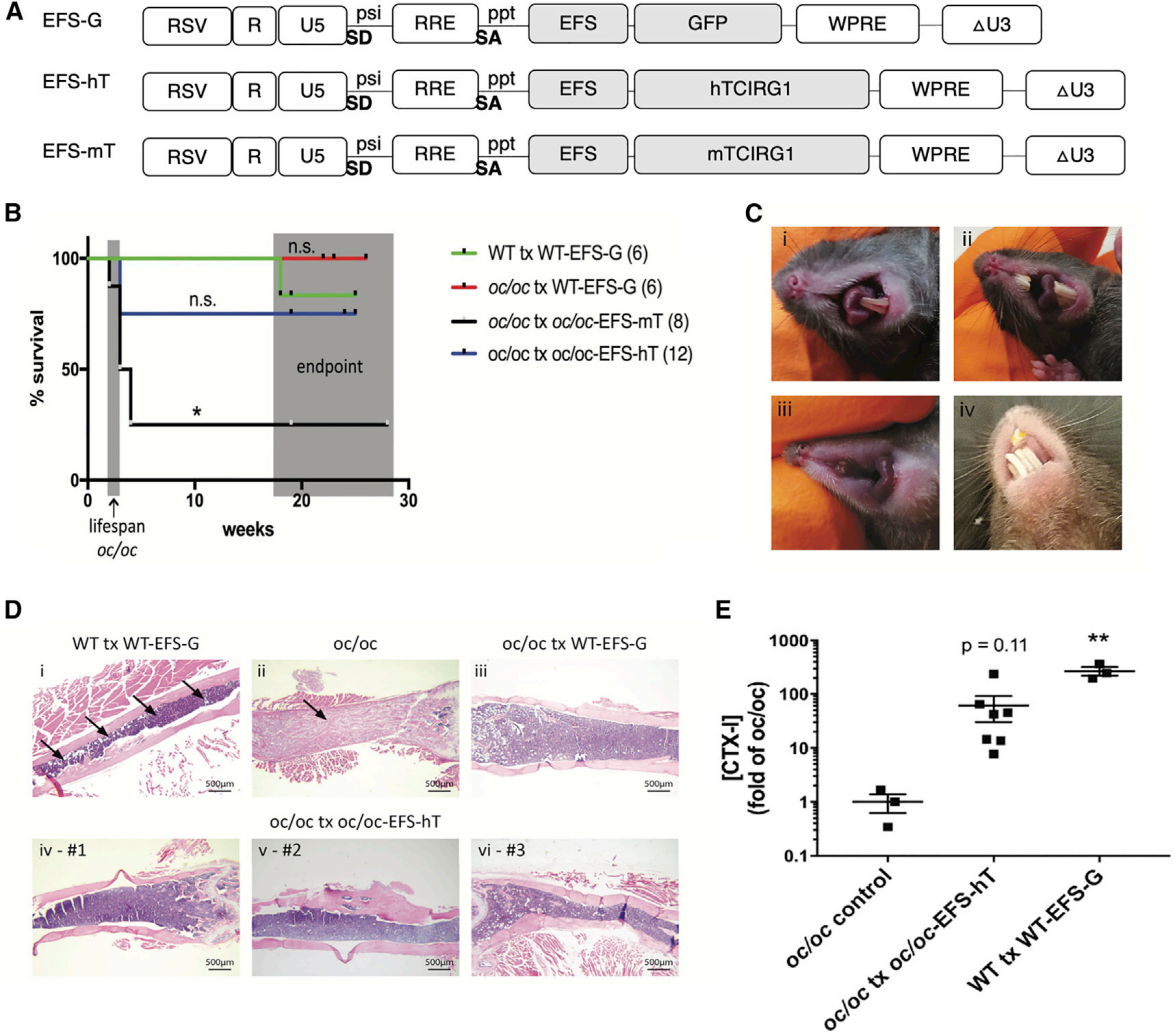
Correction of IMO phenotype in *oc/oc* mice^78^ (A) Vector constructs used in experiments. (B) Kaplan-Meier curves show long-term survival of 9/12 *oc/oc* mice transplanted with *oc/oc* FL cells transduced with EFS.hTCIRG1-LV (corresponds with EFS-hT in the figure) over time. (C and D) Reversal of the osteopetrotic bone phenotype in *oc/oc* mice after transplantation of *oc/oc* cells transduced with EFS.hTCIRG1-LV (EFS-hT in the figure) as demonstrated by histopathology. Representative images of WT transplanted (tx) WT-EFS-G (C, i), *oc/oc* tx WT-EFS-G (C, ii), *oc/oc* tx oc/oc-EFS-mT (C, iii), and *oc/oc* tx oc/oc-EFS-hT (C, iv) teeth presence/absence at the time of sacrifice. Representative images of the femur (2.5× ·objective) of a WT tx WT-EFS-G mouse (D, i) in which the arrows indicate the BM space (purple), an untreated *oc/oc* mouse (D, ii) in which the arrow indicates the osteopetrotic bone (pink), an *oc/oc* tx WT-EFS-G mouse (D, iii), and three representative surviving *oc/oc* tx oc/oc-EFS-hT mice as indicated (D, iv, v, and vi). (E) Osteoclasts differentiated from splenocytes of transplanted *oc/oc* mice express hTCIRG1 and show varying levels of bone resorption *ex vivo*. CTX-I data are shown for each individual mouse and as the mean ± SEM. Statistical significance is indicated with *p < 0.05 and **p < 0.01.

Nonclinical experiments to demonstrate phenotypic correction *in vivo* were also carried out in the mouse model of IMO.^78^ As previously demonstrated with RVs, the *oc/oc* mouse model is an optimal model in which to demonstrate proof-of-concept for EFS.hTCIRG1-LV-mediated *ex vivo* GT for the treatment of IMO patients with mutations in *TCIRG1*. To assess for phenotypic correction and prolongation of survival in this murine model of IMO, we intravenously transplanted fetal liver (FL) *oc/oc* c-*Kit*^+^ cells transduced with EFS.hTCIRG1-LV (Figure 4A) into sublethally irradiated neonatal *oc/oc* mice. 25% of *oc/oc* mice transplanted with EFS.mTCIRG1-LV-transduced *oc/oc* cells, expressing the mouse TCIRG1 protein, survived long term (Figure 4B) and did not show signs of tooth eruption (Figure 4C), although the reason for the poor rescue remains unclear.^78^ A better outcome was obtained with the human TCIRG1 protein. 75% of treated mice showed long-term survival through study completion at 19–25 weeks, exceeding that of untransplanted *oc/oc* mice, which have a lifespan of 3–5 weeks (Figure 4B). *Oc/oc* mice transplanted with EFS.hTCIRG1-LV transduced *oc/oc* cells also displayed histologic reversal of the osteopetrotic bone phenotype with evidence of tooth eruption, which is absent in *oc/oc* mice (Figures 4C and 4D). Additional evaluation of phenotypic benefit in vector-corrected osteoclasts differentiated *ex vivo* showed human TCIRG1 protein expression and increased CTX-I release relative to uncorrected osteoclasts (Figure 4E). Resorption pits on bone slices were observed for osteoclasts derived from 78% of surviving mice. In summary, this study provided additional proof-of-concept that EFS.hTCIRG1-LV constitutes an efficient and clinically applicable LV for use in the GT of severe osteopetrosis due to *TCIRG1* deficiency.^78^

Based on the very compelling body of efficacy data from the above-mentioned model systems employing the clinically applicable EFS.hTCIRG1-LV, efforts were directed toward further development including the evaluation of safety and toxicity supporting an investigational new drug (IND) application and subsequent clinical trial for IMO. Toxicology studies established the short- and long-term safety profile by demonstrating that an infusion of gene-corrected HPSCs was not associated with any physical, behavioral, biochemical, or morphologic abnormalities (data on file). Moreover, no evidence of RCLs was observed and an *in vitro* immortalization (IVIM) study demonstrated a highly reduced mutagenic risk.^53^ Furthermore, integration profiles showed no evidence of clonal dominance, leukemia, or myelodysplasia. Additional considerations regarding this favorable safety profile include the incorporation of the mammalian promoter EFS and regulatory element woodchuck hepatitis virus posttranscriptional regulatory element, WPRE, in the lentiviral construct in ongoing clinical trials for X-SCID.^39^^,^^76^^,^^79^^,^^80^ No significant therapy-related adverse events have been identified to date in these trials. Collectively, the large body of data demonstrating phenotypic correction *in vitro* and *in vivo*, coupled with a robust safety profile, supported evaluation of EFS.hTCIRG1-LV in a phase 1 clinical trial (NCT04525352).

### Conclusions and future perspectives

IMO is a devastating disorder of early childhood that presents a highly unmet medical need with limited therapeutic options. It is largely fatal in the early years of life and the only definitive therapy currently available is allogeneic HSCT.^16^, ^17^, ^18^ However, there are specific complications inherent with IMO HSCT,^19^^,^^20^ suggesting that a less toxic and more broadly applicable therapy for IMO may fulfill a substantial need in this ultra-rare and highly fatal disorder of infancy and early childhood. GT utilizing autologous hematopoietic stem and progenitor cells represents a potentially advantageous therapeutic alternative for IMO that could be performed relatively rapidly following diagnosis to create the highest opportunity for prevention or reversal of disease manifestations. An additional potential advantage of autologous hematopoietic GT is the more limited conditioning likely required to enable HSPC engraftment relative to allogeneic HSCT. Experience over recent decades has enabled establishment of consensus guidelines for HSCT in IMO, including recommendations for conditioning involving therapeutic drug monitoring (TDM)-guided busulfan in combination with fludarabine.^15^^,^^19^ Single-agent TDM-guided busulfan monotherapy has been demonstrated as an optimal conditioning strategy for autologous GT in multiple non-malignant hematopoietic disorders, and this busulfan monotherapy has been stipulated for initial GT studies in IMO.^81^ Taken together, the nonclinical studies summarized in this review indicate that the proposed autologous GT offers potential for substantive benefit including prevention of neurologic, hematologic, and other morbidities. Autologous GT may be feasible for all patients relatively rapidly after diagnosis and administered without many of the considerable toxicities associated with allogeneic HSCT.

**Figure 5:**
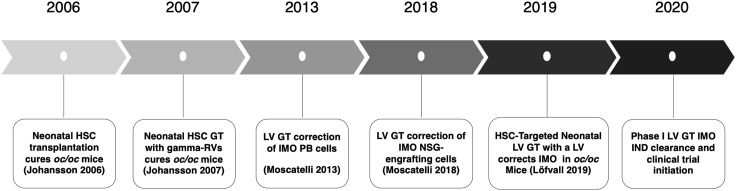
Timeline of LV gene therapy for IMO Most relevant milestone achievements toward the initiation of an LV GT clinical trial for IMO. IND, investigational new drug application.

13 years after the first experimental approach was published in 2006—targeting the correction of the *oc/oc* phenotype by HSCT—proof-of-concept in this same model was published aiming at the correction of the osteopetrotic phenotype with a clinically applicable LV, EFS.hTCIRG1-LV (Figure 5).^78^ These findings, combined with the subsequent nonclinical safety and toxicity program, have enabled a comprehensive preclinical evaluation supporting the initiation of a clinical trial evaluating GT for IMO.
